# Human bone marrow mesenchymal stem cell‐derived extracellular vesicles impede the progression of cervical cancer via the miR‐144‐3p/CEP55 pathway

**DOI:** 10.1111/jcmm.15573

**Published:** 2021-01-08

**Authors:** Qin Meng, Baofang Zhang, Yingming Zhang, Shuyan Wang, Xiaohui Zhu

**Affiliations:** ^1^ Department of Obstetrics and Gynecology Shandong Medical College Linyi P. R. China; ^2^ Department of Physical Examination The Third People's Hospital of Linyi Linyi P. R. China; ^3^ Department of Obstetrics and Gynecology Linyi People's Hospital Linyi P. R. China

**Keywords:** centrosomal protein, 55 Kd, cervical cancer, extracellular vesicles, human bone marrow mesenchymal stem cells, microRNA‐144‐3p

## Abstract

Cervical cancer is the most common gynaecological malignancy, with a high incidence rate and mortality rate in middle‐aged women. Human bone marrow mesenchymal stem cells (hBMSCs) have been implicated in the initiation and subsequent development of cancer, along with the involvement of extracellular vesicles (EVs) mediating intracellular communication by delivering microRNAs (miRNAs or miRs). This study is aimed at investigating the physiological mechanisms by which EVs‐encapsulated miR‐144‐3p derived from hBMSCs might mediate the progression of cervical cancer. The expression profiles of centrosomal protein, 55 Kd (CEP55) and miR‐144‐3p in cervical cancer cell lines and tissues, were quantified by RT‐qPCR and Western blot analysis. The binding affinity between miR‐144‐3p and CEP55 was identified using in silico analysis and luciferase activity determination. Cervical cancer cells were co‐cultured with EVs derived from hBMSCs that were treated with either miR‐144‐3p mimic or miR‐144‐3p inhibitor. Cervical cancer cell proliferation, invasion, migration and apoptosis were detected in vitro. The effects of hBMSCs‐miR‐144‐3p on tumour growth were also investigated in vivo. miR‐144‐3p was down‐regulated, whereas CEP55 was up‐regulated in cervical cancer cell lines and tissues. CEP55 was targeted by miR‐144‐3p, which suppressed cervical cancer cell proliferation, invasion and migration and promoted apoptosis *via* CEP55. Furthermore, similar results were obtained by hBMSCs‐derived EVs carrying miR‐144‐3p. In vivo assays confirmed the tumour‐suppressive effects of miR‐144‐3p in hBMSCs‐derived EVs on cervical cancer. Collectively, hBMSCs‐derived EVs‐loaded miR‐144‐3p impedes the development and progression of cervical cancer through target inhibition of CEP55, therefore providing us with a potential therapeutic target for treating cervical cancer.

## 1 INTRODUCTION

Cervical cancer, a malignancy that arises from chronic human papillomavirus (HPV) infections, ranks as the 4th most commonly diagnosed cancer among females and the 4th leading cause of cancer‐related deaths with an estimated 570 000 cases and 311 000 deaths reported across the world in 2018.^1^ Current available primary treatment approaches for patients with cervical cancer include surgery, or a concurrent chemoradiotherapy regimen supplemented with cisplatin‐based chemotherapy, beam radiotherapy and brachytherapy.^2^ The disease is not considered refractory yet the oncologic outcome of patients with recurrent and metastatic cervical cancer is still below par, despite recent achievements regarding therapeutic strategies,^3^ thus highlighting the need for developing more effective approaches against the progression of cervical cancer.

Microarray and databases conducted by both Yi *et al* and Koch *et al* have revealed that the centrosomal protein, 55 Kd (CEP55), is a clinically relevant biomarker for cervical cancer.^4^, ^5^ A functional report has demonstrated that CEP55 has the ability to reflect and indicate unfavourable clinical prognosis of patients suffering from cervical cancer,^6^ whereas the specific mechanism governing the action of CEP55 still requires further study. Intriguingly, bioinformatics analysis prior to our investigation proved that microRNA‐144‐3p (miR‐144‐3p) was a putative upstream regulatory miRNA for CEP55. Concordantly, miR‐144‐3p has been elucidated to inhibit cancer cell proliferation and promote apoptosis by targeting CEP55 in the context of prostate cancer.^7^, ^8^ miR‐144‐3p has been identified as one of the down‐regulated miRNAs in serum of patients with negative HPV16.^9^ The tumour‐suppressive action of miR‐144‐3p in cervical cancer has also been reported,^10^ whereas the underlying mechanism still remains enigmatic. Notably, miR‐144‐3p has been detected to be abundant in extracellular vesicles (EVs) derived from mesenchymal stem cells (MSCs) in association with cell growth regulation.^11^ Multiple types of cancer cells constitute tumours where MSCs, a particular population of cancer stem cells, particularly exhibit pro‐ or antitumorigenic influences on cancerogenesis.^12^, ^13^ Bone marrow‐derived MSCs (BMSCs) have been described as ‘magic bullets’ in the suppression of tumour progression, regarding their capabilities of differentiation.^14^ The paracrine functions of MSCs have been found to be partially mediated by EVs, which can shuttle miRNAs, messenger RNAs (mRNAs) and proteins involved in cell‐to‐cell communication. All of this helps suggest the promising application of MSCs‐derived EVs in mediation of cancer progression.^15^, ^16^ Although the role of miR‐144‐3p and CEP55 in cervical cancer has already been investigated, the mechanism by which EV communication affects cervical cancer cells involving the interplay between miR‐144‐3p and CEP55 is still poorly understood, highlighting a major gap in knowledge given that MSCs‐derived EVs may be of significance to the development and progression of cervical cancer. Hence, we have been suggested that the transfer of miR‐144‐3p *via* BMSCs‐derived EVs might alter the biology of recipient cervical cancer cells in mediating the development and progression of cervical cancer.

## 2 MATERIALS AND METHODS

### 2.1 Ethics statement

The study was conducted with the approval of the Ethics Committee of Shandong Medical College and was performed in strict accordance with the *Declaration of Helsinki*. Each participant signed a written informed consent prior to the study. Animal experiments were strictly designed and conducted according to the Guide for the Care and Use of Laboratory Animals published by the US National Institutes of Health. Extensive efforts were made to ensure minimal suffering of the animals used during the study.

### 2.2 In silico analysis

Cervical cancer‐related microarray was retrieved from the Gene Expression Omnibus (GEO) database (https://www.ncbi.nlm.nih.gov/geo/), followed by differential analysis using ‘limma’ package of R language. Differentially expressed genes (DEGs) were obtained with |logFoldChange|> 2 and *P* < 0.05 as the thresholds. A heat map displaying DEGs was plotted using the ‘pheatmap’ package. The expression profiles of CEP55 in cervical cancer samples and normal samples from The Cancer Genome Atlas (TCGA) were analysed by a Gene Expression Profiling Interactive Analysis data set (http://gepia.cancer‐pku.cn/index.html). The possible regulatory miRNAs of CEP55 were predicted by data sets of DIANA (http://diana.imis.athena‐innovation.gr/DianaTools/index.php?r=microT_CDS/index), mirDIP (http://ophid.utoronto.ca/mirDIP/index.jsp#r), miRSearch (https://www.exiqon.com/miRSearch) and TargetScan (http://www.targetscan.org/vert_71/). An intersection of prediction results was obtained.

### 2.3 Sample collection

We collected cancer and adjacent normal tissue samples from 60 patients (aged: 10‐58 years, with a mean age of 21.63 ± 9.70 years) with cervical cancer at the Shandong Medical College, from April 2010 to April 2013. None of these patients received either chemotherapy or radiotherapy prior to their operation. Meanwhile, 15 mL of bone marrow samples was extracted from healthy volunteers.

### 2.4 Cell culture

Human cervical cancer cell line (HeLa), human cervical squamous cell carcinoma cell line (SiHa) (Cell Bank of Shanghai Institutes for Biological Sciences, Chinese Academy of Sciences, Shanghai, China), human cervical cancer epithelial cell line (CaSki), human cervical epidermoid carcinoma cell line (ME180) (Procell Life Science & Technology Co., Ltd., Wuhan, Hubei, China) and normal cervical epithelial cell line (End1/E6E7) (Biobw Co., Ltd., Beijing, China) were cultured in an incubator at 37℃ with 5% CO_2_ following short tandem repeat identification. The cells were detached with 0.25% trypsin and inoculated in a culture plate at a concentration of 1 × 10^6^ cells/mL (adjusted by Dulbecco's modified Eagle's medium [DMEM] containing 10% foetal bovine serum [PBS]) for further use, upon reaching 80% confluence.

### 2.5 Isolation and identification of human BMSCs (hBMSCs)

hBMSCs were isolated from the donor bone marrow as previously reported^17^ and then cultured in DMEM‐Ham's F‐12 medium (F12) supplemented with 10% FBS (10099141, Gibco, Carlsbad, California, USA), and 0.2% penicillin and streptomycin (Hyclone Laboratories, Logan, UT, USA). The cells were subcultured every 3 days, and the cells at passage 3‐7 were used for subsequent experiments. The identification of hBMSC phenotype was performed by flow cytometry. Each tube was added with the fluorescence‐labelled antibodies (Abcam Inc, Cambridge, UK) to cluster of differentiation 90 (CD90) (ab226), CD105 (ab11414), CD73 (ab239246), CD34 (ab81289), CD45 (ab10558), CD44 (ab157107), CD11b (ab24874), CD14 (ab28061), CD19 (ab24936) and human leucocyte antigen‐DR (HLA‐DR) (ab20181). 10 µL of fluorescein isothiocyanate (FITC)–conjugated goat antibody to immunoglobulin G (IgG) and 10 µL of isotypical antibody of equal concentration were mixed together and allowed to react at room temperature, thus serving as an isotype negative control (NC). Antigens on the cell surface were detected using a flow cytometer and analysed by FACSuite software (BD Biosciences,San Jose, CA, USA).

### 2.6 Detection of hBMSC differentiation potential in vitro

The hBMSCs at passage 3 were detached with the concentration adjusted to 5 × 10^4^ cells/mL, followed by seeding into a 6‐well plate. The cells were allowed to grow and adhere to the coverslip, after 24 hours of seeding. Subsequently, hBMSCs were cultured in the medium for OriCell™ MSC osteogenesis, lipogenesis or cartilage differentiation (all purchased from Cyagen Company, Guangzhou, China) according to the manufacturer's instructions, followed by identification of cells using Alizarin Red S staining, oil red O staining and Alcian blue staining, respectively.^18^

### 2.7 Plasmid transfection and lentiviral transduction

Plasmid transfection was performed according to steps described in the manual of Lipofectamine 2000 reagents (11668‐019, Invitrogen, Carlsbad, WI, USA). Briefly, cells were added with miR‐144‐3p/NC mimic, miR‐144‐3p/NC inhibitor and sh‐CEP55/NC (a final concentration of 50 nmol\L) for incubation at room temperature for 5 minutes. Another 250 μL Opti‐MEM was used to dilute 5 μL Lipofectamine 2000 and was left to incubate at room temperature for 5 minutes. The above two were mixed and allowed to settle at room temperature for another 20 minutes. The mixture was then added into the cell culture plate. After culture, subsequent experiments were conducted.

LV3‐hsa‐miR‐144‐3p and package plasmids were co‐transfected into 293T cells using liposomes to construct experimental lentivirus, whereas LV3‐NC and package plasmids were co‐transfected into 293T cells using liposomes to construct NC lentivirus. The lentiviruses of LV3‐hsa‐miR‐144‐3p and LV3‐NC were obtained, following incubation in a 5% CO_2_ incubator at 37℃. The hBMSCs in the logarithmic growth phase were seeded into a 6‐well plate at a density of 1 × 10^6^ cells/mL and cultured for 48 hours in a 5% CO_2_ incubator at 37℃. The cells were subsequently screened using puromycin for at least one week to select stably transfected cell lines.

### 2.8 Dual‐luciferase reporter gene assay

The target genes of miR‐144‐3p were predicted on microRNA.org, the results of which were further verified by dual‐luciferase reporter gene assay. CEP55 3' untranslated region (3’UTR) gene fragment was artificially synthesized and introduced into pGL3‐control (Promega, Madison, WI, USA) using enzyme sites. The mutant (MUT) sites of complementary sequence of seed sequence were designed on the CEP55‐wild‐type (WT). Target fragments were inserted into pGL3‐control vector using T4 DNA ligase after restriction enzyme digestion. The luciferase reporter plasmids WT and MUT were co‐transfected with miR‐144‐3p mimic into H293T cells (Cell Bank of Shanghai Institutes for Biological Sciences), respectively. The luciferase activity was then measured.

### 2.9 Co‐culture of hBMSCs and cervical cancer cells

The hBMSCs were detached with 0.25% trypsin and cultured in low‐glucose DMEM to a cell concentration of 1.0 × 10^6^ cells/mL. Cervical cancer cells were labelled with pCDNA3.1‐GFP, whereas hBMSCs were transduced with miR‐144‐3p‐Cy3 (GenePharma, Shanghai, China). They were subsequently collected and mixed at a ratio of 1:1 after 12 hours of transfection and then seeded in a 96‐well plate (100 cells/well). Co‐culture was maintained for 2 days before flow cytometry‐based separation.

### 2.10 Isolation and characterization of EVs

The EVs in the serum were isolated and removed through the ultracentrifugation of medium/serum at 100,000 × g overnight at 4℃. The hBMSCs in viable growth conditions were cultured in a EVs‐depleted serum. The supernatant of hBMSCs at passage 3 was centrifuged at 500 × g for 15 minutes at 4℃ to remove cellular debris. The cellular debris or apoptotic bodies were further removed by centrifugation at 2000 × g for 15 minutes at 4℃, whereas large vesicles were removed by centrifugation at 10 000 × g for 20 minutes at 4℃. After the resulting supernatant was filtered through a 0.22‐µm filter and spun at 110 000 × g for 70 minutes at 4℃, hBMSCs were resuspended in PBS and subjected to another round of centrifugation (110 000 × g, 70 minutes, 4℃), followed by resuspension in 100 µL sterile PBS for subsequent experiments. The size distribution of the isolated EVs was measured using a nanoparticle tracking analysis (NTA) instrument, following the steps described in the manual. Next, 10 µL of isolated and purified EVs was left to counterstain with 3% (w/v) sodium phosphotungstate solution for 1 minute at room temperature. EVs were then observed under a transmission electron microscope (TEM).

### 2.11 Isolation and quantification of RNA

A reverse transcription quantitative polymerase chain reaction (RT‐qPCR) was employed to quantify the expression of miR‐144‐3p and mRNA expression of CEP55. Based on the related sequences provided by GenBank, the primer sequences were designed by Primer 5 software as shown in Table 1. The primers of downstream target genes were analysed by a homology analysis using BLAST software. The cells were collected, and the total RNA was extracted using a Trizol kit (Invitrogen) after transfection. Then, total RNA was reversely transcribed into complementary DNA (cDNA) according to the instructions provided by TaqMan MicroRNA Assays Reverse Transcription Primer (4427975, Applied Biosystems, Foster City, CA, USA), followed by PCR amplification. The relative expression levels of miR‐144‐3p and CEP55 were analysed by the 2^–ΔΔCt^ method with U6 serving as the loading control for miR‐144‐3p and β‐actin for CEP55.

**TABLE 1:** Primer sequences for RT‐qPCR

Target	Primer sequences (5'‐3')
miR‐144‐3p	F: 5'‐TACTGCATCAGGAACTGACTGGA‐3'
	R: 5'‐GTGCAGGGTCCGAGGT‐3'
CEP55	F: 5'‐AGTAAGTGGGGATCGAAGCCT‐3'
	R: 5'‐CTCAAGGACTCGAATTTTCTCCA‐3'
U6	F: 5'‐GCTTCGGCAGCACATATACTAAAAT‐3'
	R: 5'‐CGCTTCACGAATTTGCGTGTCAT‐3'
β‐actin	F: 5'‐CGTGACATTAAGGAGAAGCTG‐3'
	R: 5'‐CTAGAAGCATTTGCGTGGAC‐3'

Note:

Abbreviation: CEP55, centrosomal protein, 55 kD; F, forward; miR, microRNA; R, reverse; RT‐qPCR, reverse transcription quantitative polymerase chain reaction.

### 2.12 Detection of EV markers

hBMSCs‐derived EVs were resuspended in pre‐cooled lysis buffer solution containing protease inhibitor cocktail tablets (Roche Life Science, Basel, Switzerland). The lysate was dissolved in 3 × Laemmli's buffer solution, boiled for 5 minutes, analysed by 12% sodium dodecyl sulphate‐polyacrylamide gel electrophoresis (SDS‐PAGE) and transferred onto the nitrocellulose membrane. The membrane was then blocked in 0.05% Tween supplemented with 5% skimmed milk and PBS (pH = 7.4), probed with rabbit antibodies (Abcam) to ALIX (ab88743), CD63 (ab216130) and glucose‐regulated protein 94 (GRP94) (ab13509) and re‐probed with diluted horseradish peroxidase‐conjugated secondary antibody of goat anti‐rabbit IgG (ab6721, 1:10000, Abcam). The protein signal was detected by enhanced chemiluminescence (ECL) reagents (170‐8280, Bio‐Rad Laboratories Inc, Hercules, CA, USA), with Ponceau red serving as the internal reference.

### 2.13 Protein preparation and Western blot analysis

The total protein content was extracted from cells, and the protein concentration was determined by a bicinchoninic acid kit (20201ES76, Yeasen Biotech Co., Ltd., Shanghai, China). Proteins were loaded into 20 µg‐sized wells, allowed to separate by 8% SDS‐PAGE for 1 hour and blocked in 5% skimmed milk. The resulting protein was subsequently probed with diluted primary antibodies (Abcam) to β‐actin (ab8226, 1:500), cyclin‐dependent kinase 4 (CDK4) (ab137675, 1:500), Cyclin D1 (ab134175, 1:10000), E‐cadherin (ab15148, 1:500), Vimentin (ab92547, 1:1000), B‐cell lymphoma‐2 (Bcl‐2) (ab59348, 1:500), Bcl‐2‐associated protein X (Bax) (ab32503, 1:1000), CD63 and heat shock protein 70 (Hsp70). The results were visualized by an ECL instrument (Health Care, Little Chalfont, Buckinghamshire, UK) and analysed by the Image Pro Plus 6.0 image processing software (Media Cybernetics, Maryland, USA). The relative protein expression was expressed as the ratio of the grey value of protein to be tested to that of internal reference (GAPDH).

### 2.14 Transwell migration and Matrigel invasion assays

As for Transwell migration assay, 8‐µm Transwell chambers in a 24‐well plate (Corning Incorporated, Corning, NY, USA) were adopted. The apical chambers were added with 200 µL cells, whereas the basolateral chambers were added with 300 μL DMEM (Invitrogen) containing 10% FBS and penicillin/streptomycin (as chemokines), followed by 48‐hour culture at 37℃ with 5% CO_2_. Following fixation in 4% paraformaldehyde for 30 minutes, the chambers were treated with 0.2% Triton X‐100 (Sigma‐Aldrich, St. Louis, MO, USA) for 15 minutes and stained with 0.05% gentian violet for 5 minutes.

The cell invasion was detected using the same procedures as previously mentioned, except that the filter membrane was coated with 50 μL Matrigel (Sigma) in the chambers. The number of stained cells in 5 randomly selected fields was counted under an inverted microscope (XDS‐800D, Shanghai Caikang Optical Instrument Co., Ltd., Shanghai, China) to evaluate the cell capabilities of migration and invasion.

### 2.15 5‐ethynyl‐2’‐deoxyuridine (EdU) assay

Cells were left to culture in 50 µmol\L of EdU (EdU labelling/detection kit, Ribobio Co., Ltd., Guangzhou, Guangdong, China) for 12 hours, fixed with paraformaldehyde and incubated with antibodies to EdU. Next, 5 µg/mL Hoechst 33 342 was applied to stain cells for 30 minutes. The results were observed under a fluorescence microscope.

### 2.16 Colony formation assay

At 48 hours post‐cell transfection, the 6‐well plate was coated with Roswell Park Memorial Institute (RPMI) 1640 medium and allowed to stand. When solidified, the plate was added with cell suspension (0.5 mL, 2 × 10^3^ cells/mL) and resuspended by a RPMI 1640 medium formulation (GE Health Care) containing 10% FBS and 0.3% agar. The formation of cell colonies was observed after 21 days of culture at 37℃. The number of cell colonies was counted in 5 randomly selected fields in each group.

### 2.17 Flow cytometry

After transfection for 48 hours, cells were detached with trypsin (YB15050057, Shanghai Yubo Biological Technology Co., Ltd., Shanghai, China) without ethylenediaminetetraacetic acid and collected into a flow tube. Cell apoptosis was detected using the Annexin‐V‐FITC cell apoptosis detection kit (APOAF‐20TST, Sigma).

### 2.18 Xenograft tumour in nude mice

SiHa cells were dispersed into signal cell suspension after transfection. PBS was mixed with Matrigel (E1270, Sigma) at a ratio of 1:1, where cells were resuspended to a concentration of 1 × 10^6^ cells/200 μL. A total of 24 BALB/c nude mice (aged: 4‐6 weeks, weighing: 18‐20 g, Shanghai SLAC Laboratory Animal Co., Ltd., Shanghai, China) were subcutaneously inoculated with SiHa cell suspension (1 × 10^6^ cells/200 μL). When the tumour volume reached 100 mm^3^, nude mice were further injected with EVs extracted from hBMSCs‐miR‐NC or hBMSCs‐miR‐144‐3p. Every 6 tumours were selected from each group for the measurement of tumour volume and weight every day, starting from the 2nd day. The tumour volume was calculated with the formula of V = length × width^2^/2. On the 35th day, the nude mice were killed under anaesthesia, followed by the resection of tumours.

### 2.19 Immunofluorescence

The paraffin samples were cut into sections, dewaxed and dehydrated. After blocking, the sections were incubated with 50 μL primary antibodies (Abcam, UK) to CEP55 (ab170414, 1:400), CD31 (ab28364, 1:50) and Ki67 (ab15580, 1:1000) at 4℃ overnight and further incubated with secondary antibodies of goat anti‐rabbit IgG (ab150077, 1:1000, Abcam) for 30 minutes at 37℃. The sections were then developed by diaminobenzidine (DAB) (Sigma) and counterstained with haematoxylin (Shanghai Bogoo Biotech Co., Ltd., Shanghai, China). PBS was used as a NC instead of primary antibodies and the normal tissues served as a positive control. Images were captured under microscopic observation (XSP‐36, Boshida Optical Instrument Co., Ltd., Shenzhen, Guangdong, China) with 5 randomly selected high‐power fields in each section (200 ×). Every 100 cervical epithelial cells were counted. The percentage of positive cells < 10% was regarded as negative, whereas that ≥ 10% was considered positive.^19^

### 2.20 Terminal deoxynucleotidyl transferase‐mediated 2'‐deoxyuridine 5'‐triphosphate nick end labelling (TUNEL) staining

Five sections were dewaxed, hydrated and incubated with 50 μL 1% proteinase K dilution. The activity of endogenous peroxidase (POD) was blocked by the incubation with methanol solution containing 0.3% H_2_O_2_. The sections were then added with a TUNEL reaction solution and a 50 μL Converter‐POD, followed by development using 2% of DAB. The reaction was terminated by adding distilled water when the cells presented with brownish yellow nucleus. The sections were later counterstained with haematoxylin, followed by dehydration using ethanol of ascending concentrations and xylene clearing. The sections were observed under an optical microscope after being sealed with neutral gum.

### 2.21 Statistical analysis

Data analysis was performed using the SPSS 21.0 software (IBM, Armonk, NY, USA). Measurement data obeying normal distribution and homogeneity of variance were expressed as the mean ± standard deviation unless otherwise indicated. Data between two groups were compared by independent sample *t test*. Data comparisons among multiple groups were analysed by the one‐way analysis of variance (ANOVA), followed by Tukey's test. Data comparisons at different time‐points were analysed by Bonferroni‐corrected repeated measures ANOVA. A *P* < 0.05 was considered statistically significant.

## 3 RESULTS

### 3.1 mRNA expression profiles in cervical cancer

**FIGURE 1:**
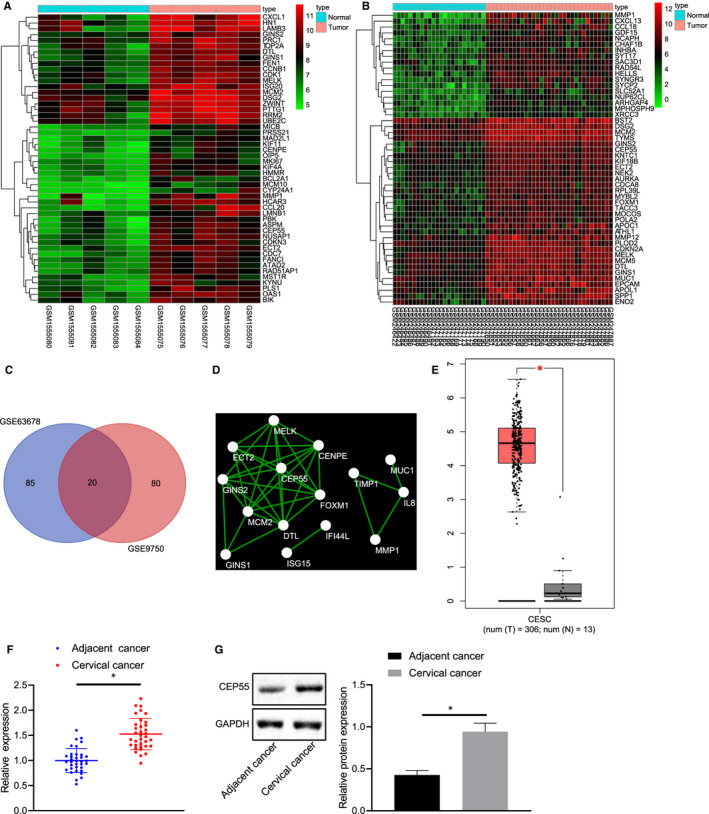
The significance of CEP55 in cervical cancer. A, B, Heat maps of significantly up‐regulated genes in cervical cancer‐related microarray data sets. The abscissa refers to the sample number, the ordinate refers to the genes, the left dendrogram represents the expression level, each cube represents the expression level of a gene in one sample, and the upper right histogram represents the colour scale. C, Venn analysis of significantly up‐regulated genes in cervical cancer‐related microarray data sets. Two circles show the significantly up‐regulated genes in cervical cancer samples in two cervical cancer‐related microarray data sets, and the middle part indicates the intersection of two sets of data. D, The interaction network of significantly up‐regulated genes in cervical cancer samples. Each circle represents a gene and the line between circles indicates the presence of interaction between two genes. E, The expression of CEP55 in cervical cancer samples (the left box plot) and normal samples (the right box plot) according to TCGA database. The abscissa refers to the sample type, and the ordinate refers to the expression level. F, Expression of CEP55 was determined by RT‐qPCR in cervical cancer and adjacent normal tissues, relative to GAPDH. The left image represents the detection results of CEP55 expression in cancer tissues, and the right represents the detection results of CEP55 expression in adjacent normal tissues. G, Representative Western blots of CEP55 protein and its quantitation (on the right side) in cervical cancer and adjacent normal tissues, relative to GAPDH. Data between cervical cancer tissues and adjacent normal tissues were compared with paired *t* test

At the initial stage of our study, cervical cancer‐related microarray data sets GSE63678 and GSE9750 were retrieved from the GEO database, followed by differential analysis. The results revealed 105 and 100 significantly up‐regulated genes in cervical cancer samples, respectively. A heat map displaying the top 50 significantly up‐regulated genes in the two microarray data sets was plotted (Figure 1A, B). An intersection of prediction results available from two microarray data sets was obtained to further screen out cervical cancer‐related genes (Figure 1C). In total, 20 significantly up‐regulated genes were detected in prediction results of both microarray data sets. An interaction network diagram was then developed for a correlation analysis (Figure 1D). CEP55 was found at the core position in the interaction network diagram of the 20 significantly up‐regulated genes, suggesting that CEP55 could probably interact with multiple up‐regulated genes. Therefore, CEP55 might play an important role in the development of cervical cancer. The expression of CEP55 was subsequently quantified in cervical cancer samples and normal samples that were available from the TCGA database (Figure 1E). The results indicated that CEP55 was significantly highly expressed in cervical cancer samples. For further verification, we collected cervical cancer tissues and adjacent normal tissues, followed by conducting RT‐qPCR and Western blot analysis. The results revealed that the expression of CEP55 was much higher in cervical cancer tissues than that in adjacent normal tissues (Figure 1F, G), which was just in line with bioinformatics analysis in microarray data sets related to cervical cancer.

### 3.2 CEP55 was highly expressed in cervical cancer cell lines that contributed to the progression of cervical cancer

**FIGURE 2:**
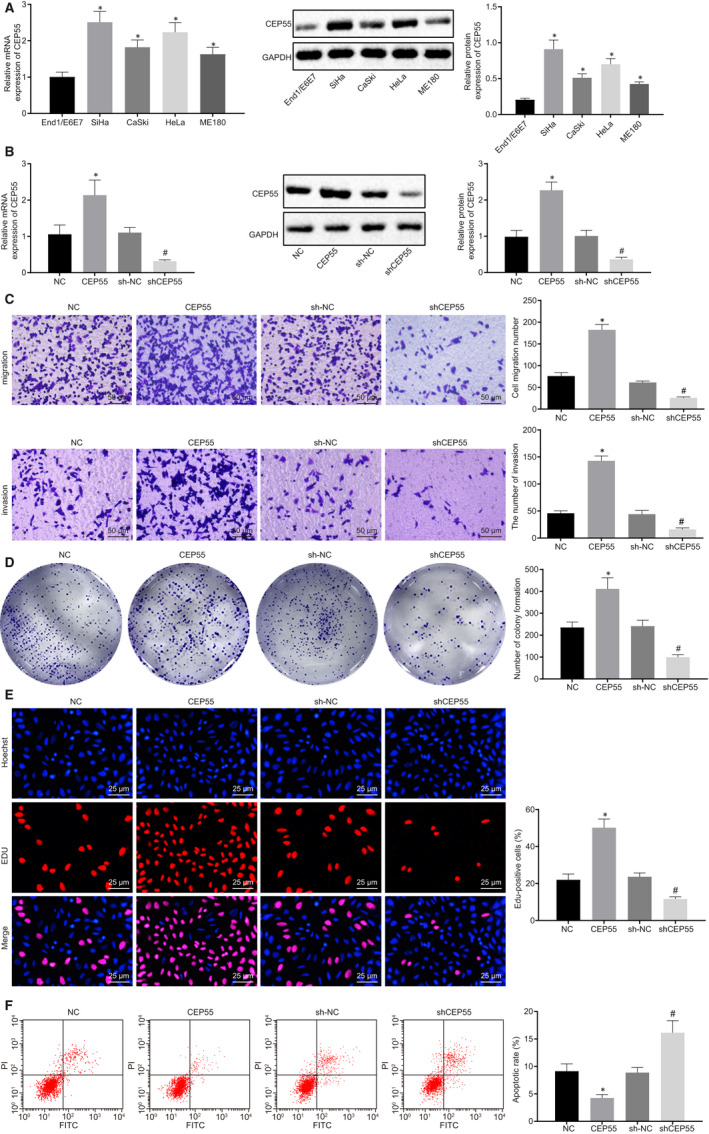
High expression of CEP55 contributes to tumorigenesis of cervical cancer. A, mRNA and protein expression of CEP55 was determined by RT‐qPCR and Western blot analysis in normal cervical epithelial cell line End1/E6E7, and cervical cancer cell lines HeLa, CaSki, SiHa and ME180, normalized to GAPDH. B, mRNA and protein expression of CEP55 was determined by RT‐qPCR and Western blot analysis in SiHa cells, normalized to GAPDH. C, SiHa cell migration and invasion were detected by Transwell assay (scale bar = 50 µm). D, Colony formation of SiHa cells was detected by colony formation assay. E, SiHa cell proliferation was detected by EdU assay (scale bar = 25 µm). F, SiHa cell apoptosis was detected by flow cytometry. Data comparison was analysed by independent sample *t test* between two groups and by one‐way ANOVA among multiple groups, followed by Tukey's test. *P < 0.05 versus the End1/E6E7 cell line or SiHa cells treated with NC. ^#^ *P* < 0.05 *versus* SiHa cells treated with sh‐NC. Data are shown as the mean ± standard deviation of three technical replicates

Following culture, the expression profiles of CEP55 in normal cervical epithelial cell line End1/E6E7 and cervical cancer cell lines, HeLa, CaSki, SiHa and ME180, were determined by RT‐qPCR and Western blot analysis (Figure 2A). CEP55 expression was elevated in cervical cancer cell lines HeLa, CaSki, SiHa and ME180, compared to that of the normal cervical epithelial cell line End1/E6E7, among which the SiHa cell line exhibited the highest expression of CEP55. Therefore, the SiHa cell line was selected for subsequent experiments for transfection of NC, CEP55, sh‐NC and shCEP55. RT‐qPCR and Western blot analysis were conducted to measure the resulting expression changes of CEP55. The results revealed that the treatment of shCEP55 led to a diminished CEP55 expression, whereas the treatment of CEP55 led to an obvious elevation of CEP55 expression, when compared with the corresponding NCs (Figure 2B). Subsequent gain‐ and loss‐of‐function assays were performed to evaluate cell migration and invasion by Transwell assay (Figure 2C), clone‐forming ability by colony formation assay (Figure 2D), proliferation by EdU assay (Figure 2E) and apoptosis by flow cytometric analysis (Figure 2F). The presence of shCEP55 corresponded to weakened cell migration, invasion, clone‐forming ability and proliferation, along with strengthened cell apoptosis, whereas a contrary changing tendency was induced in the presence of CEP55. Taken together, overexpressed CEP55 potentiated the cellular capabilities of proliferation, migration, invasion and colony formation while inhibiting the capability of cell apoptosis, all of which could be reversed with the down‐regulated expression of CEP55 expression in cervical cancer.

### 3.3 miR‐144‐3p was poorly expressed in cervical cancer cell lines and tissues acting as an anti‐oncomiR in vitro

**FIGURE 3:**
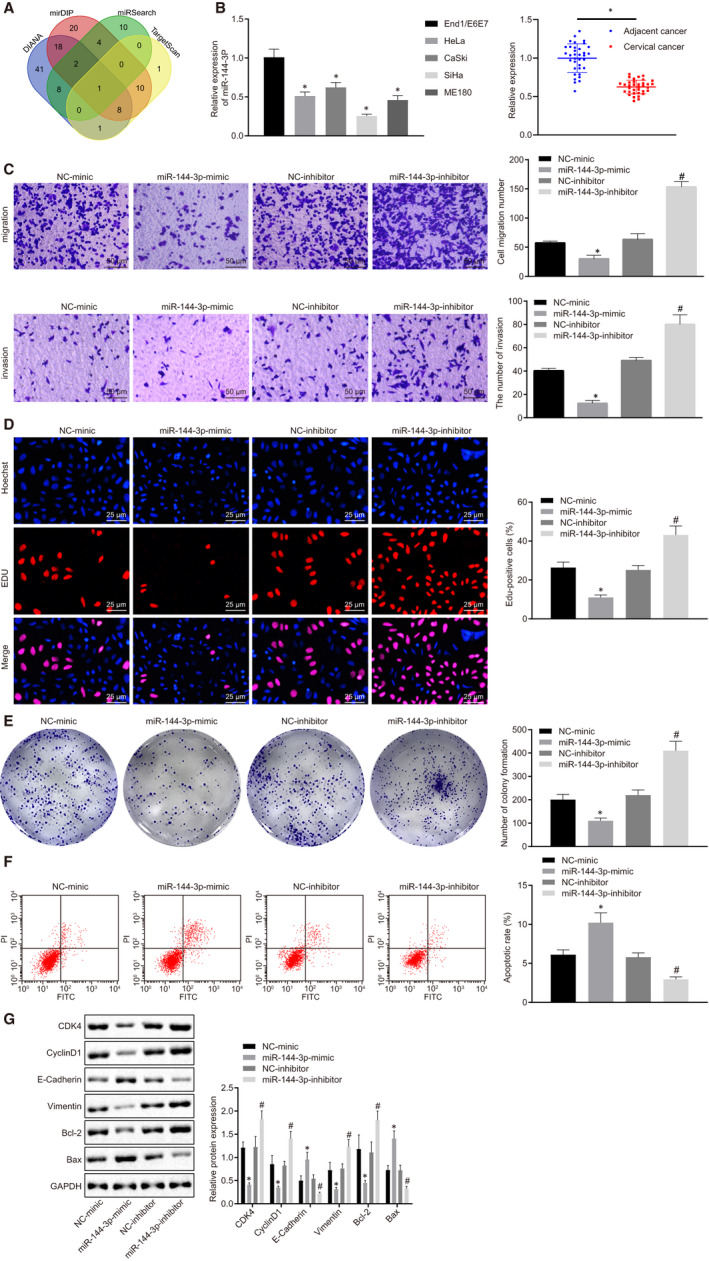
High expression of miR‐144‐3p exerts inhibitory effects on cervical cancer tumorigenesis. A, Putative miRNAs that might regulate CEP55. The 4 ellipses indicate prediction results from 4 databases, and the middle part represents the intersection. B, Expression of miR‐144‐3p was determined by RT‐qPCR in normal cervical epithelial cell line End1/E6E7, and cervical cancer cell lines, HeLa, CaSki, SiHa and ME180, (on the right side) as well as cervical cancer and adjacent normal tissues (on the left side), relative to U6. C, SiHa cell migration and invasion were detected by Transwell assay (scale bar = 50 µm). D, SiHa cell proliferation was detected by EdU assay (scale bar = 25 µm). E, Colony formation of SiHa cells was detected by colony formation assay. F, SiHa cell apoptosis was detected by flow cytometry. G, Representative Western blots of CDK4, Cyclin D1, E‐cadherin, Vimentin, Bcl‐2 and Bax proteins and their quantitation in SiHa cells, normalized to GAPDH. Data comparisons were analysed by an independent sample *t test* between two groups and by one‐way ANOVA among multiple groups, followed by Tukey's test. *P < 0.05 versus SiHa cells treated with NC. ^#^ *P* < 0.05 versus SiHa cells treated with sh‐NC. Data are shown as the mean ± standard deviation of three technical replicates

To further explore the upstream regulatory mechanism of CEP55, miRNAs that could regulate CEP55 were predicted by databases, including DIANA. According to the intersection of prediction results of 4 databases, the only one miRNA found was miR‐144‐3p (Figure 3A). The expression patterns of miR‐144‐3p in normal cervical epithelial cell line End1/E6E7 and cervical cancer cell lines, HeLa, CaSki, SiHa and ME180, were determined by RT‐qPCR. The miR‐144‐3p expression was reduced in cervical cancer cell lines HeLa, CaSki, SiHa and ME180, compared with that of the normal cervical epithelial cell line, End1/E6E7. In addition, miR‐144‐3p expression was also down‐regulated in cervical cancer tissues compared with that of adjacent normal tissues (Figure 3B).

The SiHa cell line with the lowest miR‐144‐3p expression was transfected with NC mimic, miR‐144‐3p‐mimic, NC inhibitor and miR‐144‐3p inhibitor to investigate the effects of aberrantly expressed miR‐144‐3p in cervical cancer cells. As detected by the Transwell assay (Figure 3C), EdU assay (Figure 3D), colony formation assay (Figure 3E) and flow cytometric analysis (Figure 3F), cell malignant behaviours, including migration, invasion, clone‐forming ability and proliferation, were enhanced by the miR‐144‐3p inhibitor but were suppressed by the miR‐144‐3p mimic, whereas cell apoptosis exhibited contrasting changes. A Western blot analysis was performed to determine the protein levels of related proteins (CDK4, Cyclin D1, E‐cadherin, Vimentin, Bcl‐2 and Bax). The results suggested that the delivery of miR‐144‐3p mimic led to lower expressions of CDK4, Cyclin D1, Vimentin and Bcl‐2, but increased expressions of E‐cadherin and Bax in cells. However, the delivery of miR‐144‐3p inhibitor resulted in contradicting changes. To conclude, miR‐144‐3p exerted inhibitory effects on cervical cancer cell proliferation, migration and invasion while promoting apoptosis in vitro.

### 3.4 miR‐144‐3p exerted inhibitory effects in cervical cancer cells by targeting CEP55

**FIGURE 4:**
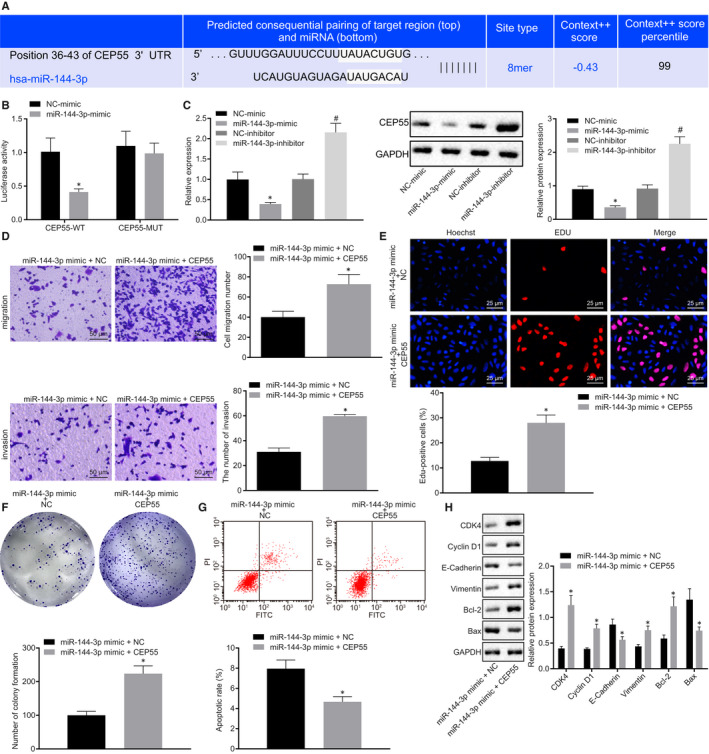
CEP55 is required for the tumour‐suppressive effects of overexpressed miR‐144‐3p in cervical cancer. A, Putative miR‐144‐3p binding sites in the 3'UTR of CEP55 mRNA in the bioinformatics website (http://mirdb.org/). B, miR‐144‐3p binding with the 3'UTR of CEP55 mRNA confirmed by dual‐luciferase reporter gene assay. C, mRNA and protein expression of CEP55 was determined by RT‐qPCR and Western blot analysis in SiHa cells, normalized to GAPDH. D, SiHa cell migration and invasion were detected by Transwell assay (scale bar = 50 µm). E, SiHa cell proliferation was detected by EdU assay (scale bar = 25 µm). F, Colony formation of SiHa cells was detected by colony formation assay. G, SiHa cell apoptosis was detected by flow cytometry. H, Representative Western blots of CDK4, Cyclin D1, E‐cadherin, Vimentin, Bcl‐2 and Bax proteins and their quantitation in SiHa cells, normalized to GAPDH. Data comparison was analysed by independent sample *t test* between two groups. *P < 0.05 versus SiHa cells treated with NC mimic or miR‐144‐3p mimic + NC. ^#^ *P* < 0.05 versus SiHa cells treated with NC inhibitor. Data are shown as the mean ± standard deviation of three technical replicates

The bioinformatics prediction website (http://mirdb.org/) predicted that misR‐144‐3p targeted CEP55 by binding to the 3’UTR of CEP55 mRNA (Figure 4A). Dual‐luciferase reporter gene assay was conducted for further verification. As shown in Figure 4B, the luciferase signal of CEP55‐WT was suppressed in cells transfected with miR‐144‐3p mimic (*P* < 0.05), yet that of CEP55‐MUT remained unaffected (*P* > 0.05), indicating that miR‐144‐3p could specifically bind to CEP55. To further unravel the detailed regulatory mechanism, CEP55 expression was determined by RT‐qPCR in SiHa cells treated with miR‐144‐3p mimic and miR‐144‐3p inhibitor. The results revealed that the expression of CEP55 was decreased in cells upon treatment with miR‐144‐3p mimic, but increased in response to miR‐144‐3p inhibitor treatment (Figure 4C), thus suggesting an inverse relationship between CEP55 and miR‐144‐3p.

Subsequently, miR‐144‐3p mimic + NC and miR‐144‐3p mimic + CEP55 were delivered into SiHa cells, followed by the assessment of cellular behaviour. According to the results of the Transwell assay (Figure 4D), EdU assay (Figure 4E), colony formation assay (Figure 4F) and flow cytometric analysis (Figure 4G), the cellular capabilities of migration, invasion, proliferation and colony formation were suppressed, whereas apoptosis was promoted in cells by the treatment of miR‐144‐3p mimic alone when compared with that of the combined treatment of miR‐144‐3p mimic + CEP55. The protein levels of related proteins (CDK4, Cyclin D1, E‐cadherin, Vimentin, Bcl‐2 and Bax) were then measured by Western blot analysis in cells, the results of which revealed down‐regulated expression of CDK4, Cyclin D1, Vimentin and Bcl‐2, along with up‐regulated expression of E‐cadherin and Bax in cells in the presence of miR‐144‐3p mimic + NC in comparison to those in cells treated with miR‐144‐3p mimic + CEP55 (Figure 4H). Given the aforementioned findings, the tumour‐suppressive effects of miR‐144‐3p on cervical cancer cells could be restored by up‐regulated CEP55.

### 3.5 hBMSCs were able to secret EVs

**FIGURE 5:**
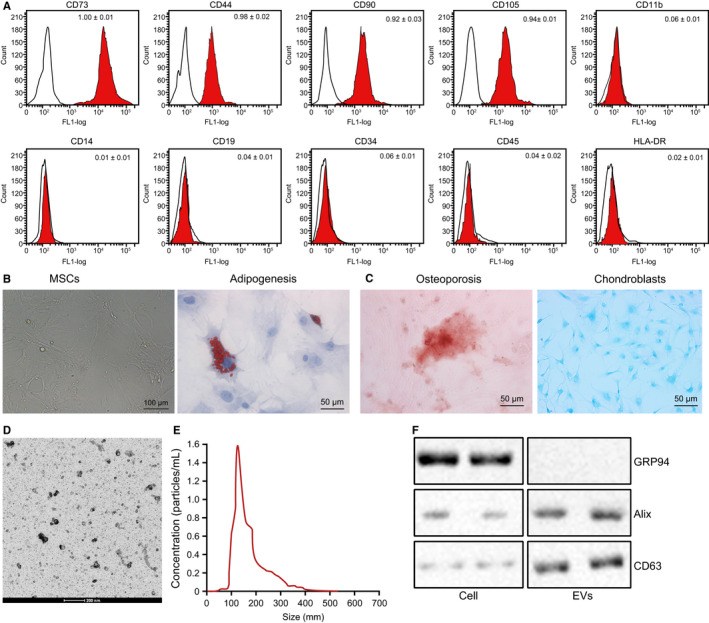
hBMSCs are successfully isolated. A, Expression of surface antigens of hBMSCs was identified by flow cytometry. B, Morphology of hBMSCs under an optical microscope (scale bar = 100 µm). C, Adipogenic (left), osteogenic (middle) and chondrogenic (right) differentiation of hBMSCs were detected by oil red O staining, Alizarin red staining and Alcian blue staining (scale bar = 50 µm). D, Morphology of EVs under a TEM (scale bar = 200 nm). E, Size distribution of EVs was detected by NTA. F, Representative Western blots of EV marker proteins and their quantitation in hBMSCs

After hBMSCs were isolated at passage 3, the surface antigen was identified by flow cytometry using FITC‐labelled mouse anti‐human antibodies to CD29, CD34, CD44, CD45, CD71 and HLA‐DR. The positive expressions of CD73 (proportion of 1.00 ± 0.01), CD44 (proportion of 0.96 ± 0.02), CD90 (proportion of 0.92 ± 0.03), and CD105 (proportion of 0.94 ± 0.01) accounted for more than 95%, whereas CD34 (proportion of 0.06 ± 0.01), CD45 (proportion of 0.01 ± 0.02), CD11b (proportion of 0.04 ± 0.01), CD14 (proportion of 0.06 ± 0.01), CD19 (proportion of 0.04 ± 0.01) and HLA‐DR (proportion of 0.02 ± 0.01) exhibited negative results (Figure 5A), fulfilling all characteristics of stem cells. hBMSCs were elongated or spindle shaped and grew in clusters with dense whirlpool‐like distributions (Figure 5B), based on microscopic observation. According to osteogenic, adipogenic and chondrogenic differentiation assays (Figure 5C), hBMSCs harboured great potential to differentiate into osteoblasts, adipocytes and chondroblasts. EVs were then isolated from hBMSCs, followed by the observation under a TEM (Figure 5D). The hBMSCs‐derived EVs were round or oval in irregular sizes and had diameters ranging from 50 to 200 nm (Figure 5E), based on NTA analysis. In addition, Western blot analysis showed that the expression of ALIX and CD63 was detected in EVs, in the absence of GRP94 expression (Figure 5F). These findings demonstrated that EVs were successfully isolated from hBMSCs, which harboured the great potential of multi‐differentiation.

### 3.6 hBMSCs‐derived EVs carried miR‐144‐3p into cervical cancer cells

**FIGURE 6:**
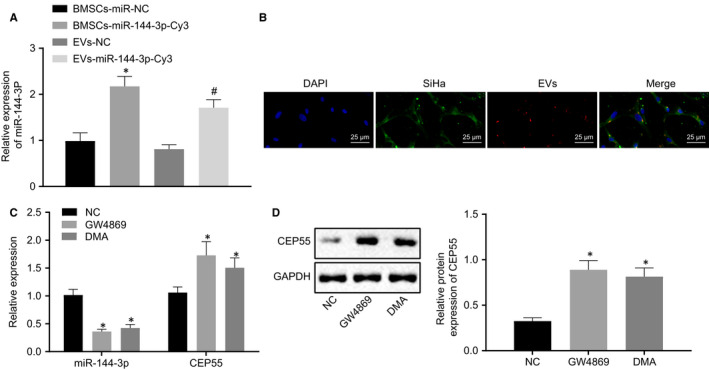
miR‐144‐3p can be transferred into cervical cancer cells by EVs derived from hBMSCs. A, Expression of miR‐144‐3p was determined by RT‐qPCR in cells, relative to U6. B, miR‐144‐3p‐Cy3 in hBMSCs and cervical cancer cells under the fluorescence microscope (scale bar = 20 µm). C, Expressions of miR‐144‐3p and CEP55 were determined by RT‐qPCR in cells, relative to U6 and GAPDH, respectively. D, Representative Western blots of CEP55 protein and its quantitation in cells, normalized to GAPDH. Data comparison was analysed by independent sample *t test* between two groups and by one‐way ANOVA among multiple groups, followed by Tukey's test. *P < 0.05 versus hBMSCs treated with miR‐NC or NC. ^#^ *P *< 0.05 versus EVs treated with NC. Data are shown as the mean ± standard deviation of three technical replicates

We then elucidated whether EVs could function as vehicles shuttling miR‐144‐3p into cervical cancer cells to regulate the expression of CEP55, so as to mediate the development of cervical cancer. The expression of miR‐144‐3p in cervical cancer cells after co‐culture was determined by RT‐qPCR (Figure 6A), the results of which revealed that miR‐144‐3p expression was increased in cells in the presence of hBMSCs‐miR‐144‐3p‐Cy3 and EVs‐miR‐144‐3p‐Cy3, but decreased in the presence of EVs‐NC in comparison with that of hBMSCs‐miR‐NC. Under the guidance of a fluorescence microscope (Figure 6B), red fluorescence was observed in hBMSCs and cervical cancer cells successfully transfected with miR‐144‐3p‐Cy3, whereas no fluorescence was detected in cells without transfection. The percentage of fluorescent cells in total cells was then counted and compared at the exact same location. The results showed that fluorescent cells accounted for approximately 60% in hBMSCs and 80% in cervical cancer cells, showing that miR‐144‐3p could be transferred into cervical cancer cells *via* EVs derived from hBMSCs.

EV inhibitors (GW4869 and DMA) were added to investigate the effects of EVs. RT‐qPCR (Figure 6C) and Western blot analysis (Figure 6D) were performed for the quantification of miR‐144‐3p and CEP55 in miR‐144‐3p‐hBMSCs following co‐culture. The results revealed that the addition of GW4869 and DMA led to lower miR‐144‐3p expression and higher mRNA and protein expressions of CEP55 in cells. The above‐mentioned findings elucidated that miR‐144‐3p expression was suppressed in cervical cancer cells by EV inhibitors.

### 3.7 miR‐144‐3p in hBMSCs‐derived EVs exerted inhibitory effects in cervical cancer cells

**FIGURE 7:**
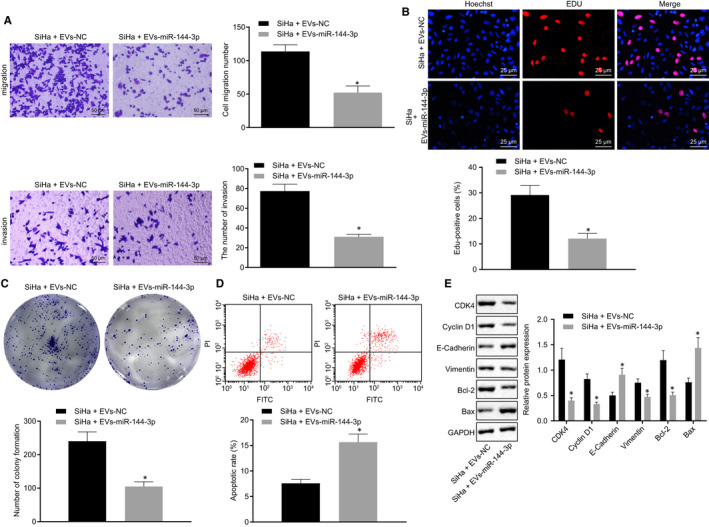
EVs‐encapsulated miR‐144‐3p secreted from hBMSCs acts as a tumour suppressor in cervical cancer. A, SiHa cell migration and invasion were detected by Transwell assay (scale bar = 50 µm). B, SiHa cell proliferation was detected by EdU assay (scale bar = 25 µm). C, Colony formation of SiHa cells was detected by colony formation assay. D, SiHa cell apoptosis was detected by flow cytometry. E, Representative Western blots of CDK4, Cyclin D1, E‐cadherin, Vimentin, Bcl‐2 and Bax proteins and their quantitation in SiHa cells, normalized to GAPDH. Data comparison was analysed by independent sample *t test* between two groups. *P < 0.05 versus SiHa cells treated with EVs‐NC. Data are shown as the mean ± standard deviation of three technical replicates

EVs were isolated from hBMSCs‐miR‐NC and hBMSCs‐miR‐144‐3p and added into cervical cancer cells, assigned as the SiHa + EVs‐NC and SiHa + EVs‐miR‐144‐3p groups. Transwell assay (Figure 7A), EdU assay (Figure 7B), colony formation assay (Figure 7C) and flow cytometric analysis (Figure 7D) were performed to evaluate the resulting cellular malignant behaviours, followed by Western blot analysis for further verification. In SiHa cells co‐cultured with EVs in the presence of miR‐144‐3p, cell migration, invasion, proliferation and colony formation were suppressed, whereas apoptosis was promoted accompanied by lowered levels of CDK4, Cyclin D1, Vimentin and Bcl‐2, along with higher levels of E‐cadherin and Bax in cells. Therefore, these findings elaborated the tumour‐suppressive effects on miR‐144‐3p in EVs derived from hBMSCs in cervical cancer cells.

### 3.8 Combined treatment of miR‐144‐3p and hBMSCs suppressed tumour growth of cervical cancer in vivo

**FIGURE 8:**
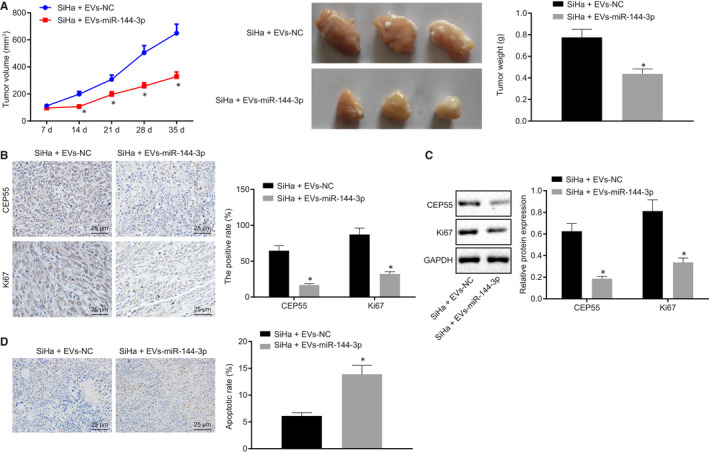
miR‐144‐3p exerts synergistic tumour‐suppressive effects in cervical cancer in vivo with hBMSCs. A, The growth of cervical cancer xenograft tumour in nude mice is measured every 7 d. B, Positive expression of CD31, CEP55 and Ki67 proteins detected by immunohistochemistry in tumour tissues (scale bar = 25 µm). C, Representative Western blots of CEP55 and Ki67 proteins and their quantitation in tumour tissues, normalized to GAPDH. D, Cell apoptosis was detected by TUNEL assay in tumour tissues (scale bar = 25 µm). Data comparison was analysed by independent sample *t test* between two groups. *P < 0.05 versus nude mice treated with EVs‐NC. Data are shown as the mean ± standard deviation of three technical replicates

Lastly, in order to validate the effects of above‐mentioned axis in vivo, SiHa cells were subcutaneously injected into the left axilla of nude mice, followed by the injection of EVs into the tumour site of mice belonging to hBMSCs‐miR‐NC (EVs‐NC) and hBMSCs‐miR‐144‐3p (EVs‐miR‐144‐3p) groups. The tumorigenicity and tumour metastasis of mice were evaluated. The results indicated that since the 21st day, the tumour growth rate of mice was accelerated in the presence of EVs‐NC when compared with that in the presence of EVs‐miR‐144‐3p (Figure 8A). The expressions of CEP55 and Ki67 were detected by immunohistochemistry (Figure 8B) and Western blot analysis (Figure 8C), the results of which revealed down‐regulated expressions of CEP55 and Ki67 in tumour tissues of nude mice bearing SiHa cells co‐cultured with miR‐144‐3p‐treated EVs. Cell apoptosis in xenograft tissues was elevated in the presence of EVs‐miR‐144‐3p, as measured with TUNEL assay (Figure 8D). In conclusion, miR‐144‐3p, in combination with hBMSCs, might exert inhibitory effects on the tumour growth of cervical cancer in vivo.

## 4 DISCUSSION

MSCs, characterized by self‐renewal ability and multi‐differentiation potential into osteocytes, chondrocytes and adipocytes, have been recognized to play a critical role in managing malignancies.^20^, ^21^ In this study, we explored the underlying mechanism of hBMSCs‐derived EVs and their involvement of interplay between miR‐144‐3p and CEP55 in the tumorigenesis of cervical cancer. Data obtained in our study demonstrated that miR‐144‐3p could be incorporated into EVs derived from hBMSCs and transferred into cervical cancer cells, where miR‐144‐3p exerted anti‐tumour properties through the target inhibition of CEP55 by suppressing malignant cellular biological behaviours in vitro, and inhibiting the tumorigenicity of cervical cancer cells in vivo.

We found that CEP55 was up‐regulated in cervical cancer cells and tissues, thus contributing to the development and progression of cervical cancer. Largely in agreement with our findings, the expression of CEP55 has been reported to be 9‐fold higher when compared with adjacent normal tissues, which is associated with advanced tumour stage and a higher risk of lymph node metastasis in patients with cervical cancer.^6^ A similar expression profile of CEP55 has also been indicated in multiple types of cancers, including non–small‐cell lung cancer and breast cancer.^22^, ^23^ An analysis of the circular RNA‐miRNA‐mRNA network has revealed CEP55 as a potential mRNA implicated in the pathology of cervical cancer.^4^

Furthermore, an inverse relation between CEP55 and miR‐144‐3p was identified in our study and miR‐144‐3p was found to be expressed at a low level in cervical cancer cells and tissues. The targeting relationship between CEP55 and miR‐144‐3p has been validated in prostate cancer cells.^7^, ^8^ The expression of miR‐144‐3p has been observed to be lost in clinically‐collected tumour tissues and cells of cervical cancer, whereas increased expression of miR‐144‐3p exerts inhibitory effects on cancer cell growth both in vitro and in vivo.^10^ In our experiment, we delivered miR‐144‐3p mimic into SiHa cells for exploration purposes and discovered suppressed cell proliferation, migration, invasion and colony formation of SiHa cells, accompanied by promoted apoptosis as demonstrated by diminished levels of CDK4, Cyclin D1, Vimentin and Bcl‐2, as well as elevated levels of E‐cadherin and Bax. Up‐regulation of CDK4 and Cyclin D1 has been elucidated to be indicative of promoting effects induced by the receptor for activated protein kinase C on cervical cancer cell growth.^24^ The down‐regulation of CDK4 helps to suppress cell proliferation in human cervical cancer.^25^ E2F8, a direct target of miR‐144, significantly stimulates proliferation of papillary thyroid cancer cells by up‐regulating Cyclin D1,^26^ suggesting the adverse correlation of miR‐144 with Cyclin D1. Consistently, decreased Vimentin and increased E‐cadherin have been found as hallmarks of suppressive effects exerted by the silencing of forkhead box protein A1 on occurrence of epithelial mesenchymal transition in HeLa cells.^27^ Amplified expression of miR‐144 leads to decreased Vimentin and elevated E‐cadherin in lung adenocarcinoma cells.^28^ Likewise, the overexpression of miR‐143 results in an increased level of Bax and a decreased level of Bcl‐2 level in HeLa cells, thus serving as the circumstantial evidence of anti‐cancer effects.^29^ The function of overexpressed miR‐144‐3p in SiHa, following the delivery of miR‐144‐3p mimic, was observed to be counteracted by up‐regulated CEP55.

Subsequently, miR‐144‐3p was found to be loaded in EVs isolated from hBMSCs and further transferred into recipient cells (cervical cancer cells) where the anti‐tumour action of miR‐144‐3p was validated. The tumour microenvironment has a key role in tumorigenesis involving exchange of EVs.^30^ EVs have the capability of transporting bioactive molecules from donor cells to recipient cells implicated in intracellular signalling modulation.^31^ miRNAs encapsulated in EVs have been recognized to influence the biological function of recipient cells.^32^ For instance, the EV mechanism by which tumour‐related miRNAs are transferred from MSCs to breast cancer cells has been functionally characterized.^33^ The functional roles of miR‐144‐3p loaded in EVs derived from hBMSCs were provided by nude mouse models of cervical cancer.

**FIGURE 9:**
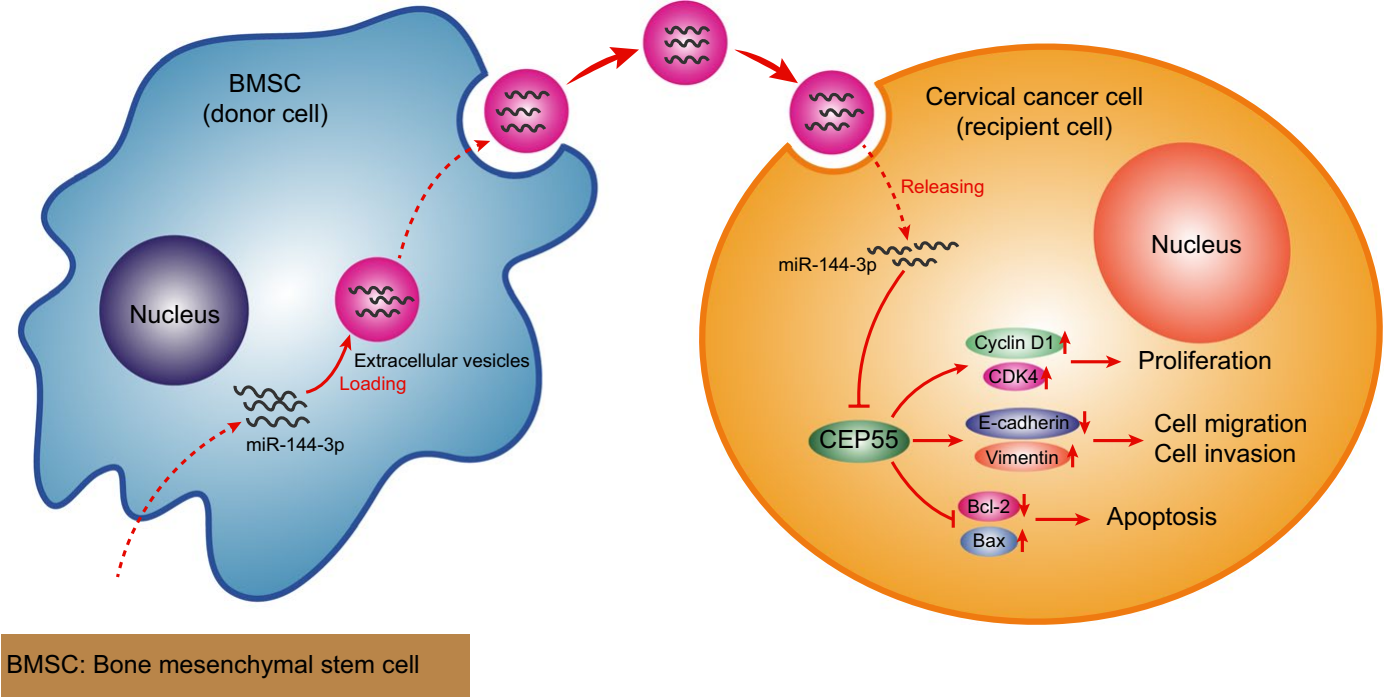
A graphical illustration of the function and mechanism of miR‐144‐3p in combination with hBMSCs. miR‐144‐3p can be transferred into cervical cancer cells *via* hBMSCs‐derived EVs. In cervical cancer cells, miR‐144‐3p exerts inhibitory effects on cervical cancer cell proliferation, migration and invasion, the mechanism of which depends on targeting relationship with CEP55

To sum up, our study demonstrated that the transfer of miR‐144‐3p *via* hBMSCs‐derived EVs altered the biology of recipient cervical cancer cells by curbing cell proliferation, migration, invasion and clonogenicity, while inducing apoptosis, all of which lead to a decreased propensity in the development and progression of cervical cancer (Figure 9). Our findings pave way for the development of effective therapeutic strategies for the treatment cervical cancer. However, multiple paracrine factors may be potentially involved in hBMSCs‐mediated effects, and the effects of EVs may be dose‐dependent, requiring more scrupulous and logical studies in the future to support more promising clinical treatments for cervical cancer.

## CONFLICT OF INTEREST

None.

## AUTHORS' CONTRIBUTIONS

Xiaohui Zhu involved in the conceptualization, investigation, methodology, supervision, writing original draft, reviewing and editing the manuscript. Baofang Zhang involved in the conceptualization, methodology, validation, reviewing and editing the manuscript. Yingming Zhang involved in the data curation, formal analysis, supervision, visualization, reviewing and editing the manuscript. Shuyan Wang involved in the data curation, formal analysis, software, reviewing and editing the manuscript. Qin Meng involved in the resources, validation, writing original draft, reviewing and editing the manuscript.

## Data Availability

The data sets used and/or analysed during the current study are available from the corresponding author on reasonable request.
